# Angiogenesis and Minimal Residual Disease in Patients with Acute Myeloid Leukemia

**Published:** 2020-04-01

**Authors:** Pardis Nematollahi, Azar Baradaran, Zahra Kasaei Koopaei, Hamidreza Sajjadieh

**Affiliations:** 1 Department of Pathology, Cancer Prevention Research Center, Isfahan University of Medical Science, Isfahan, Iran; 2 Department of Pathology, School of Medicine, Shiraz University of Medical Sciences, Shiraz, Iran; 3 Department of Pathology, Isfahan University of Medical Science, Isfahan, Iran; 4 Department of Internal Medicine, Isfahan University of Medical Science, Isfahan, Iran

**Keywords:** Acute myeloid leukemia, Blood vessels, Minimal residual disease

## Abstract

**Background:** Acute myeloid leukemia (AML) is the most prevalent acute leukemia in adults. Bone marrow angiogenesis is crucial for pathogenesis of leukemia, and increasing bone marrow Mean Vascular Density (MVD) and level of angiogenesis factors are seen in patients with AML. Higher level of bone marrow MVD is associated with poor prognosis of AML according to previous studies. The present study aimed to compare bone marrow MVD in AML patients and controls and evaluate the relation between bone marrow MVD and number of residual blast cells after AML treatment.

**Materials and Methods:** This study is a longitudinal study on AML patients who were admitted to Omid hospital. The bone marrow biopsies of patients with AML and patients with normal diagnosis –as control group- were taken from archives of pathology laboratory. Immunohistochemistry staining was used for all specimens by using thrombomodulin markers for calculating MVD. Flow cytometry findings of AML patients were assessed for percent of minimal residual disease (MRD) after AML treatment in AML patients group.

**Results:** In this study, 27 AML patients and 24 healthy individuals with mean age of 40.92±15.13 years were evaluated, of whom 56.86% were male. The mean bone marrow MVD was significantly higher in AML patients than controls. The mean bone marrow MVD was significantly higher in males and there was insignificant reverse correlation between bone marrow MVD and MRD. About 59.3% of AML patients had response to treatment and there was no significant relationship between MVD and response to treatment.

**Conclusion:** Bone marrow MVD was higher in AML patients than controls and there was no remarkable relationship between bone marrow MVD and MRD and response to treatment.

## Introduction

 Leukemia is a hematological neoplasm induced by uncontrolled proliferation of immature leukocytes^1^. Acute myeloid leukemia (AML) is most prevalent in adults, and one of the most prevalent cause of death in the United States. With a low survival rate, although leukemia is considered a treatable disease these days, it is rarely treated^2^. The exact pathogenesis of leukemia and factors modulating cell proliferation in leukemia have not yet been fully known^3^. 

Bone marrow angiogenesis is crucial to pathogenesis of leukemia^4^. Bone marrow angiogenesis is the formation of new blood vessels, playing an important role in pathophysiology of hematologic malignancies ^5^^, ^^6^. The mean bone marrow micro-vascular density (MVD), recently recognized as a prognostic value^7^, shows neoplastic cell angiogenesis capable of predicting prognosis and invasion of disease. Increasing bone marrow MVD and level of angiogenesis factors seen in patients with different types of hematological malignancies like AML, was associated with the severity of leukemia ^8^^,^^9^. Decrease in bone marrow MVD and angiogenesis factors were seen after AML remissions^10^. There is evidence that higher levels of bone marrow MVD is associated with poor prognosis of AML^9^.

On the other hand, one of the best ways to assess the chemotherapeutic response and risk of relapse in acute leukemia patients is the minimal residual disease (MRD) measurement. Nowadays, multicolor flow cytometry is considered a specific and sensitive method to investigate MRD^11^^,^^12^.

Previous studies evaluating bone marrow MVD in patients with AML and comparing it with the controls, as well as studies evaluating the relationship between angiogenesis and response to treatment are, to our knowledge, limited. Accordingly, this study aims to compare the bone marrow MVD in AML patients with the controls and to evaluate the relationship between bone marrow MVD and the number of residual blast cells after AML treatment in Isfahan. 

## MATERIALS AND METHODS

 This study is longitudinal, conducted on new cases of AML patients admitted to Omid Hospital affiliated to Isfahan University of Medical Sciences (IUMS) between 2013 and 2017. The bone marrow biopsies of patients with AML processed with paraffin-wax blocks were taken for thrombomodulin immunostaining to evaluate angiogenesis. The inclusion criteria for these specimens were as follows: 1) presence of adequate tissue on paraffin-wax block for immunohistochemistry (IHC) staining and 2) presence of the 28^th^ day flow cytometry findings of specimens about the proportion of minimal residual disease (MRD) by multicolor flow-cytometry (four colors) considering the aberrant expression of markers after AML treatment. Where the 28^th^ day flow cytometry specimens were diluted, the specimens were excluded from the study. The bone marrow biopsies of patients with normal diagnosis in paraffin-wax blocks were taken from the archives of pathology laboratory and treated as control groups. The bone marrow biopsies of the control groups were from the patients with anemia or vitamin B12 deficiency. 

All the biopsy specimens were firstly fixed in paraformaldehyde, and then embedded in paraffin and decalcified with EDTA. Hematoxylin and eosin-stained slides were evaluated by a skillful pathologist to confirm diagnosis. 4 µm-thickness sections of bone marrow biopsy specimens were used for IHC staining to identify the endothelial cells with antihuman thrombomodulin antibody (Anti-Thrombomodulin antibody [141C01] (ab33513), abcam®). 

**Figure 1:**
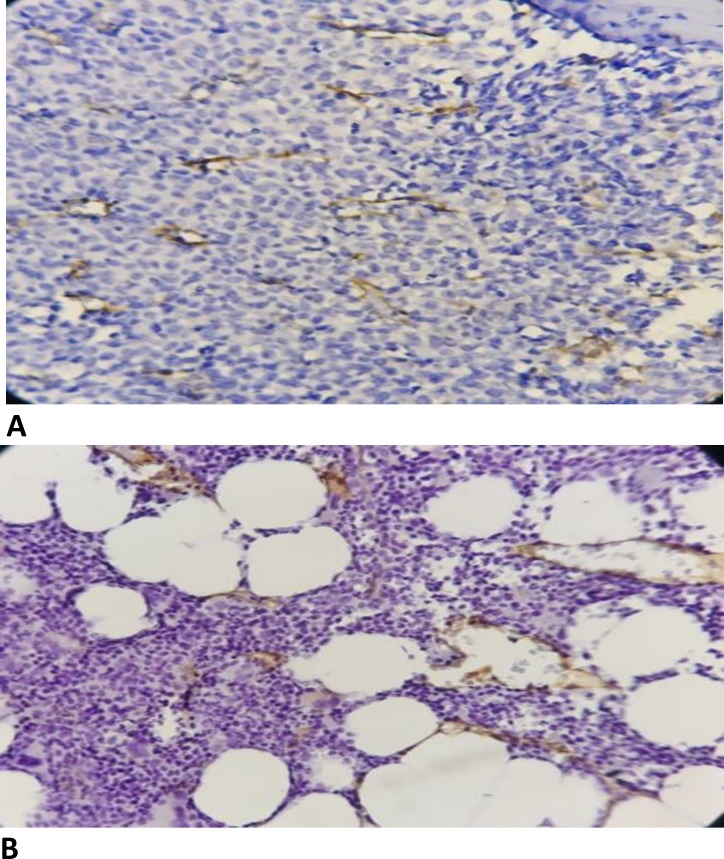
Thrombomodulin immunohistochemically staining of bone marrow biopsy of patients with AML (A) and control (B), X400 original magnification

All the specimens were evaluated by two pathologists in blinded manner using a light microscope as follows: first, at ×100 magnification, three fields with a maximum number of vessels were selected as hot spots. Then, at ×400 magnification, the field was set to cover the maximum number of vessels within the hot spots. Next, the vessels were counted in all the hotspots. Each single cell or cell accumulation with or without lumen formation previously colored with IHC staining was treated as blood vessel and the mean number of the cells from the three locations was reported as bone marrow MVD. Figure 1 shows the immunohistochemistry staining with thrombomodulin.

**Blast Counting and Response to Chemotherapy**

The data were extracted from the patients' medical records as follows: age, gender, type of disease (M3 or non-M3 AML patients), and flow cytometry (FCM) findings about the amount of the minimal residual disease (MRD) after induction chemotherapy in AML patients group based on antigen aberrancy or leukemia associated with the phenotype compared with the normal or regenerating marrow on the first presentation. Four-color FCM along with Dako and Exbio Company antibodies were performed on the bone marrow aspirates, with the panel comprising eight tubes and up to 800,000 events per tube acquired. The MRD was considered as the population showing the deviation from the normal expression of antigens. 

**Statistics analysis**

The data were input to SPSS 22 (SPSS crop. Chicago, IL, USA) and then analyzed. To report the quantitative and qualitative data, the mean ± standard deviation and number or proportions were used, respectively. To analyze the data from the independent t-test, Chi-square and Pearson Correlation Coefficient tests were used. A two-sided α-level of 0.05 was used to assess the statistical significance. The study was approved by the Ethics Committee of IUMS. 

## Results

 In this study, 27 AML patients and 24 healthy individuals were evaluated. The mean age of the participants in the AML group and controls were 40.89±16.35 and 40.96±13.76 years, respectively (P =0.99). About 70.37% (n=19) of the patients in the AML group and 79.16% (n=19) of the patients in the controls were under 50 (P =0.47). About 66.7% (n=18) in the AML group and 45.8% (n=11) in the control group were male (P-value=0.13). In the AML group, 29.5% (n=7) had M3 type of AML and 74.1% (n=20) had non-M3 type of AML. After induction chemotherapy, morphological assessment of bone marrow aspiration smears revealed that 71/4 % of patients achieved AML remission and 29/6 % of them had blast more than 5%. 

The mean bone marrow MVD was significantly higher in the AML patients than in the controls (AML patients: 29.33±10.9, controls: 11.88±4.31, P<0.001). The mean bone marrow MVD was higher in patients with M3 type of AML than in those with non-M3 types of AML, but it was not significant (P-value=0.21). There was no significant relationship between the mean bone marrow MVD and age (P =0.7). The mean bone marrow MVD was significantly higher in males than in females (24.34±12.47 vs. 17±10.72, P

 =0.032) (Table1).

**Table1:** The mean MVD in patients and control groups

**Variable**		**MVD** **Mean (SD)**	**P-value**
Group	AML	29.44 (10.9)	<0.001*
	Control	11.88 (4.31)	
Age	Under 50	20.79 (11.72)	0.7
	Above50	22.31 (12.47)	
Gender	Male	24.34 (12.47)	0.032
	Female	17 (10.72)	
Type of AML	M3	34 (10.08)	0.21
	Non-m3	27.88 (10.97)	

* The mean MVD was significantly different between AML patients and controls

**Figure 2:**
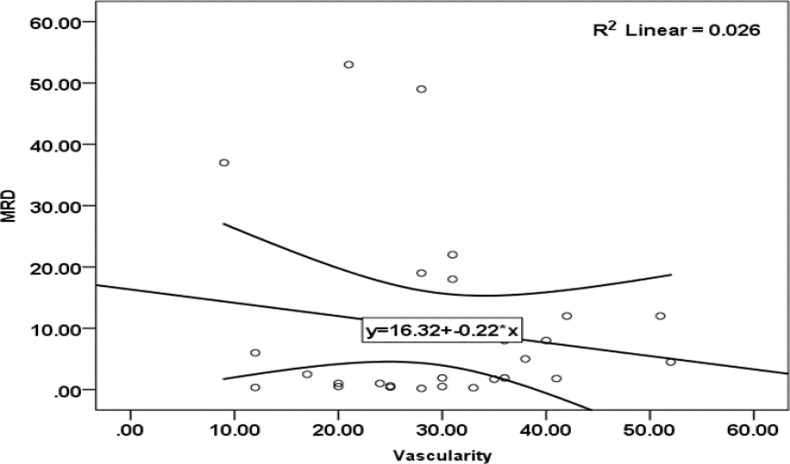
The correlation between MRD and MVD

As shown in Figure 2, the Pearson Correlation Coefficient Test showed an insignificant reverse correlation between the bone marrow MVD and MRD (P-value=0.42). 

None of the patients showed a blast of under 0.1%; about 33/3% (n=9) of them showed a blast range of 0.1-1 %, and finally about 66/7% (n=18) showed a blast of over 1%. There was no significant relationship between the MRD and age, gender and type of AML (P: 0.135, 0.58 and 0.18). The mean MVD was 32.27±11.23 and 27.5±10.59 in patients with a blast of over and under 1%, respectively (P =0.27).

## Discussion

 This study evaluated the mean bone marrow MVD in AML patients and controls. The mean between the bone marrow MVD and MRD showed no significant relationship between the bone marrow MVD and MRD, a higher bone marrow MVD in AML patients as compared to the control group and males as compared to females. The mean MRD was significantly higher in M3 types of AML.

The mean bone marrow MVD was higher in AML patients than in the controls in this study. There are similar studies that have evaluated angiogenesis in AML patients. Padro et al. showed that the mean angiogenesis was 2-3 fold higher in patients with AML than in the controls and the patients with complete remission of diseases had lower rates of angiogenesis than those with partial remissions. This study used MRD as a factor for evaluating the response to treatment, but it did not evaluate the relationship between angiogenesis and MRD^10^. Another study showed a higher rate of angiogenesis in patients with AML than in the controls, and like this study, there was no significant difference in the angiogenesis in the different types of AML^13^. Another study conducted on 30 AML patients and 30 healthy individuals for evaluation of the bone marrow MVD in these two groups reported that the mean bone marrow MVD was higher in AML patients^14^. This study showed a higher bone marrow MVD in males than in females. However, other similar studies did not show a significant relationship between angiogenesis and gender^15^. It is probable that the higher bone marrow MVD in males in this study was due to the higher number of male patients in AML group. 

This study, like previous studies, showed no significant relationship between the bone marrow MVD and MRD or response to treatment. One study on AML patients having evaluated the bone marrow MVD before, during, and after AML treatment, reported that there was no significant relationship between the bone marrow MVD and disease relapses, but this study did not use MRD as a factor for evaluating the response to treatment and relapses^7^. Another study reported that the increased level of bone marrow MVD was associated with poorer prognosis of disease in AML patients, but it did not use MRD^9^ for prognosis evaluation. Chemotherapy in patients with AML inhibits angiogenesis inducing endothelial cell apoptosis^16^. It also reduces residual blast cells. Further, there was a hypothesis that there might be a relationship between angiogenesis and MRD that was not supported in this study.

For further evaluation of the relationship between angiogenesis and MRD, more studies are needed with greater sample sizes, including other angiogenesis factors excluding bone marrow MVD. 

This study showed lower MRD in M3 AML patients than in non-M3 AML patients, which may be the result of different types of treatment. Recent advances in the treatment of patients with the M3 type of AML showed a remarkable increase in the treatment outcomes^17^. Since 1990, using a medication like all-trans-retinoic acid in chemotherapies has significantly increased the survival in m3 AML patients with 20% decrease in the relapse rate^18^. There is some evidence that modern treatments can cause complete remission in M3 patients^19^. 

This study has its strengths and limitations. One strength of this study was calculating the bone marrow MVD in patients as an angiogenesis factor, used widely in different previous studies. Another strength was measuring the blast number using multicolor flow cytometry, which is sensitive and specific method to evaluate MRD. On the other hand, this study suffers from limitations. One limitation of this study was the relatively small sample size, which may restrict the generalization of results to the general population. Another limitation was failure to use serum angiogenesis factors evaluated along with the bone marrow MVD like previous studies. Further studies are needed with larger sample sizes, including other angiogenesis factors or molecular parameters to evaluate the exact relationship between angiogenesis and MRD. 

## CONCLUSION

 In conclusion, the bone marrow MVD was higher in AML patients than in the controls and there was no remarkable relationship between the bone marrow MVD, MRD, and response to treatment.
